# Unraveling the Link Between GERD and Hypertension: Coincidence or Causal Connection?

**DOI:** 10.1002/jgh3.70234

**Published:** 2025-07-21

**Authors:** Maryam AlAlawi, Sanjiv Mahadeva

**Affiliations:** ^1^ Division of Gastroenterology, Department of Medicine, Faculty of Medicine University Malaya Kuala Lumpur Malaysia

Gastroesophageal reflux disease (GERD) and hypertension (HTN) are prevalent conditions that pose significant health challenges globally.

The prevalence of GERD is increasing, with pooled rates varying by geographic region. Several risk factors—such as age, alcohol consumption, BMI, pregnancy, education level, marital status, and medication use—contribute to its rise. As a result, GERD is now recognized not just as a gastrointestinal disorder but as a condition with systemic implications affecting multiple body systems [1]. GERD has also been observed to occur alongside metabolic syndrome and cardiovascular disease [2]. GERD symptoms can range from heartburn, dry cough, vomiting, hoarseness, and chest pain to difficulty swallowing, among others. This wide variety of symptoms can make diagnosis challenging or delayed, particularly because they may also indicate other gastrointestinal conditions such as eosinophilic esophagitis, functional dyspepsia, and gastroparesis [3]. Although lifestyle modifications and dietary changes are the primary treatments for GERD, some patients do not respond to these approaches and may require medications like antacids or proton pump inhibitors. In more severe cases, endoscopic or surgical interventions may be necessary.

Hypertension is the most significant preventable risk factor for cardiovascular disease (CVD) and overall mortality globally [4]. This is primarily due to an aging population, unhealthy diets high in salt, and insufficient physical activity. Hypertension may be discovered incidentally or, in some cases, may present with symptoms such as headaches or complications like myocardial infarction, stroke, kidney failure, and other related conditions. The growing availability of various antihypertensive medications may have played a role in stabilizing or reducing the prevalence of hypertension in recent decades.

Although previous studies indicate a potential association between GERD and hypertension, the results have been inconsistent, with some studies reporting conflicting outcomes.

Hypertension was found to be linked to a 1.5‐fold increased risk of developing GERD [5]. Considering hypertension as an inflammatory condition has prompted research into the involvement of vascular and other organ inflammation in its development. One suggested mechanism involves systemic inflammatory responses associated with GERD [6].

Two studies published in JGH Open have recently explored this relationship further. Bushi et al. [7] conducted a systematic review and meta‐analysis to examine the relationship between GERD and hypertension, aiming to clarify the findings of earlier studies. The evidence was derived from observational studies with varying designs, including retrospective cohort, cross‐sectional, and prospective approaches; however, this may limit the ability to establish a definitive association. Incorporating Randomized Controlled Trials (RCTs) would enhance the strength of the findings by helping to establish a causal relationship between GERD and hypertension. To ensure transparency and reproducibility in selecting relevant studies, the PRISMA flowchart was employed for study selection—complementing the use of diverse observational designs and reinforcing the methodological rigor of the review despite the absence of randomized controlled trials. Moreover, this systematic review did not clearly specify the diagnostic criteria used for GERD and hypertension in the included studies. Establishing standardized diagnostic criteria would have helped reduce variability and enhance comparability across studies. The findings underscore the complexity and variability of the relationship across different populations, with some studies reporting a strong association, including odds ratios as high as 6.53. However, the broad range of risk ratios (0.573–3.260) and confidence intervals (0.992–1.922) indicates inconsistency in the results, highlighting the need for further investigation. Despite the strengths of the study, a significant publication bias was detected, as indicated by an LFK index of −3.18. Additionally, high heterogeneity was observed (*I* ^2^ = 99% for prevalence and *I* ^2^ = 76% for risk ratio), reflecting variations in study populations, methodologies, and diagnostic criteria, which limit the generalizability of the results. Conducting subgroup analyses based on factors such as age, gender, geographic location, and study design could help identify sources of heterogeneity and enhance the interpretation of findings. Moreover, asymmetry observed in the DOI and funnel plots suggests potential distortion of the pooled effect estimates. The paper also highlighted the shared risk factors between GERD and HTN; however, it did not thoroughly explain how these confounding factors were controlled for across the studies. Building on this, although the primary aim was to explore the link between the two conditions, incorporating patient‐centered outcomes—such as quality‐of‐life measures—could have significantly enhanced the study's relevance and clinical applicability.

In the second study by Li et al. [8], an intriguing exploration using bidirectional Mendelian randomization (MR) analysis of the potential causal relationship between gastroesophageal reflux disease (GERD) and hypertensive disorders of pregnancy—specifically preeclampsia (PE) and gestational hypertension (GH). The study leveraged genetic data from publicly available sources, such as GWAS and the FinnGen Biobank, to reduce the influence of confounding variables and mitigate the risk of reverse causation. Similar to the previous study, this paper also showed heterogeneity in its findings, as indicated by the MR analysis (*p* < 0.05), likely due to inconsistent case definitions across the GWAS datasets. Implementing standardized case definitions and enhancing the quality of GWAS datasets could help reduce such heterogeneity in future research. Likewise, large‐scale randomized controlled trials are needed to validate these findings and evaluate whether treating GERD can effectively reduce the risk of hypertensive disorders in pregnancy. Although the study included a large sample size, it was limited to individuals of European ancestry, which restricts the generalizability of its findings to other ethnic groups and regions. Broadening the population scope in future research would enhance the applicability of the results across diverse populations. The paper also recognizes the inconsistent findings from previous studies concerning the role of GERD treatments, such as proton pump inhibitors (PPIs), in preventing hypertensive disorders of pregnancy (HDP), which may undermine confidence in their clinical applicability [9, 10].

In conclusion, current evidence points to a potential association between GERD and hypertension; however, several limitations still hinder a clear understanding of the exact nature of this relationship. These limitations do not diminish the value of the studies compared to earlier research on the association between GERD and hypertension. However, by addressing these gaps, future studies can yield more conclusive findings, minimize bias, and generate practical insights for clinical care and public health strategies.

## Conflicts of Interest

Prof. Sanjiv Mahadeva is an Editorial Board member of JGH and a co‐author of this article. To minimize bias, he was excluded from all editorial decision‐making related to the acceptance of this article for publication.

## Data Availability

Research data are not shared.
